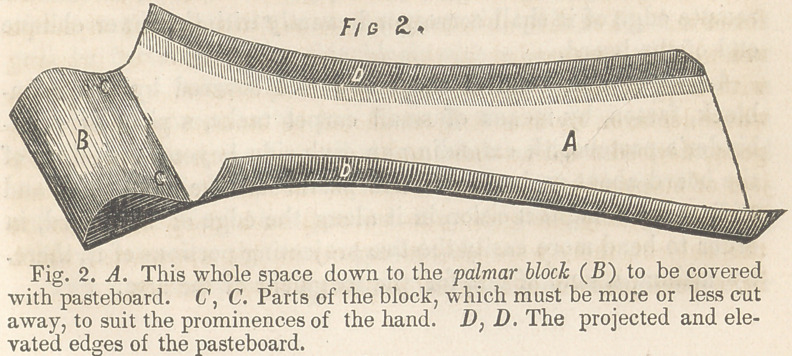# Fractures of the Lower End of the Radius, and on Their Management

**Published:** 1852-03

**Authors:** H. Bond

**Affiliations:** Philadelphia


*Fractures of the lower end of the Radius, and on their Management.* By H. Bond, M. D., Philadelphia. (Read before the Philadelphia College of Physicians and published in their "Transactions.")

I ask the attention of the College to some remarks upon fractures of the radius, especially to those which occur near its carpal extremity. These injuries have a strong claim to our attention, both on account of the *frequency of their occurrence*, and on account of the *imperfect success* so often attending their treatment.

According to the statistics of M. Goyraud, these constitute one-third of the whole number of fractures. Dupuytren assigns to them, if not always the first, at least the second or third rank in point of frequency. Vidal declares that the fracture of the radius is certainly more frequent than that of any other bone. According to reports of cases in the Hotel Dieu, Paris, for four years, they constituted one-tenth of all the fractures.

The statistics of the Pennsylvania Hospital, by Drs. Norris and Wallace, do not furnish the desired information on this subject; for they only report the fractures of the arm, without even discriminating between the arm and forearm. More precision in classification would have much enhanced the worth of these very valuable statistics. But it was not in their power to remedy defects in the records of the institution. The number of fractures treated in this hospital from 1751 to 1800 was 197, of which 34 were of the arm, which is about 17 per cent., or a little more than one-sixth. From 1800 to 1829, 868 fractures, of which 200 were of the arm, which is about 23 per cent. From 1830 to 1839 inclusive (10 years) 946 fractures (exclusive of a few cases of ununited fractures, where the part affected is not specified,) of which 250 were fractures of the arm, which is more than 26 per cent. [See *Amer. Journ. Med. Sciences*, xxiii. 260; i. new ser., p. 324.] It is probable that at least one-eighth, if not one-sixth, of the whole number were fractures of the radius.

The relative frequency of these accidents in any district will vary according to the pursuits of the people, and according to the climate and season. A storm of sleet, covering the streets and fields with ice, seldom fails to produce numbers of such cases; I am informed that one storm of this kind, not long since, besides the cases occurring in private practice, brought nine cases of fracture of the forearm into the Pennsylvania Hospital.

In a note at the end of the "Remarks on Fractures," by Dr. Peirson, is an elaborate report of the cases of fracture admitted into the Massachusetts General Hospital, from 1821 to 1840. This report exhibits a remarkable variation, in regard to the relative frequency of the fracture of different bones, from other reports that have come under my observation. It deserves an explanation by the officers of that institution.

The following is the summary of the cases detailed.

"A table showing the proportionate number of cases of each bone fractured in 367 hospital cases.

| | Cases. | |
|---|---|---|
| Fractures of the Tibia and Fibula . . . . | 95 | |
| " " Femur . . . . . . | 69 | |
| " " Tibia singly . . . . . | 38 | |
| " " Fibula singly . . . . . | 20 | |
| | — | 222 |
| " " Humerus . . . . . | 18 | |
| " " Radius singly . . . . . | 9 | |
| " " Radius and Ulna . . . . | 6 | |
| " " Ulna singly . . . . . | 4 | |
| | — | 37 |
| " " Cranium . . . . . . | 19 | |
| " " Clavicle . . . . . . | 26 | |
| " " Ribs . . . . . . | 24 | |
| " " Cervix Femoris . . . . . | 10 | |
| " " Sternum . . . . . . | 2 | |
| Miscellaneous cases: Scapula, Cervix Humeri, Carpal and Tarsal Bones, Condyles, Olecranon, and Acromion Processes | 27 | |
| Total | 367 | |

"This table shows there are six times as many fractures of the leg and thigh as of the arm and forearm; fracture of the leg being the most frequent accident, then the thigh, then the forearm, and lastly the arm."

As to the success attending the treatment of fractures of the radius, I have met with no statistical reports entitled to consideration, and I think we shall in vain search journals, hospital reports, monographs, and systematic works, for an accurate report of a series of such cases. There is a good reason for this; for, as I believe, few or no practitioners could render such a report, if made with minute fidelity, as would be flattering to their skill, or speak well for the perfection of our art. According to my observation, there is no fracture, except the neck of the thigh bone, where there is so often a failure to accomplish a perfect cure, as that of the lower end of the radius. Practitioners may tell us that, although they have often *seen* deformed wrists, and rigid or anchylosed hands, *they* have had good success—have always cured; yet a critical examination of their cases would often show that there remained either more or less deformity of the radius or the wrist, a more or less complete loss of the use of the hand, or an extremely tedious recovery, much more tedious than occurs ordinarily in other factures.

To show that I am not dealing in exaggerations, or offering opinions unsustained by the observations of others, I cite two very good American authorities. Dr. Peirson, in the work already referred to, says, "Notwithstanding the greatest care in the adjustment and treatment of fractures of the forearm, many instances of deformity will occur. Our pathological museums present an immense variety of irregular consolidations of the radius and ulna, which must have seriously interfered, during life, with their appropriate functions." In a very valuable paper of Dr. J. Rhea Barton, published in the *Medical Examiner* for 1840, he says, "I do not know any subject on which I have been more frequently consulted than on deformities, rigid joints, inflexible fingers, loss of the pronating and supinating motions, and neuralgic complaints, resulting from injuries of the wrist and the carpal extremity of the forearm; one or more of these evils having been left not merely as a temporary inconvenience, but as a permanent consequence."

It may be asked, are these unfavorable results, so opprobious to the profession, inevitable? or may they not very often be attributed to a defect in the manner of treating them? The deformity is, I believe, almost always unnecessary. The fragments are generally without difficulty placed in apposition, in their normal relation to each other; there is no powerful counteracting force tending to derange them, when the hand is kept in a position to put the muscles in repose; and the deformity results either from the inadequacy of the apparatus, or from defective skill in its application. There may be a few comparatively rare exceptions to this, as where the lower fragment of the radius is crushed, and when this condition is complicated with a fracture of the ulna. But there is an evil, as clearly specified by Dr. Barton, frequently succeeding these fractures, which is a much greater calamity to the patient than any slight or moderate deformity of the radius or wrist. This is a stiffness, inflexibility of the hands and fingers, which destroys their use or impairs it in proportion to the rigidity. This rigidity is sometimes unavoidable, as in cases where there has been severe contusion of the soft parts, and when occurring in elderly persons of a rheumatic or gouty diathesis.

I propose to examine the mode of treating these fractures, as now commonly practised in our hospitals, and as taught by the most recent American and English authors.

It is unnecessary to state in detail to the members of this College what this practice is. I may briefly say that it consists in the use of two long straight splints, with compresses or cushions, and bandages. The palmar splint extends from the elbow down to the extremities of the fingers. Some, however, do not allow this to extend below the second joints of the fingers. The dorsal splint extends sometimes only to the extremity of the metacarpe. When this dressing is applied, the longitudinal axis of the forearm will be continuous, or parallel, with that of the hand.

There are several objections to this mode of dressing the fracture, which I will attempt to point out. In the first place, it violates what ought to be regarded as a surgical canon in the treatment of fractures, viz., to adopt such a position as will put all the muscles, acting on the part, as much in repose, as free from tension, as possible; so that the least counteracting force will be required. 2d. The constrained position of the hand demands tighter bandaging, in order to prevent derangement of the fragments by paralyzing or subduing the muscles that are rendered tense by the position assumed. 3d. This constrained position and tight bandaging greatly increase the danger of that protracted or permanent rigidity of the hands and fingers which is a too frequent result of those injuries. 4th. This mode of dressing, by long, straight splints, not only increases the danger that it will result in rigidity, but that, when it does occur, the hand will be left unsightly, inconvenient, or useless. 5th. There is another objection to it, which will be regarded by the surgeon as of more or less importance, according as he is actuated more or less by the feelings of humanity. I refer to the distress or discomfort which must result from a constrained position and the force applied to maintain it.

The muscles that act on the hand are least tense, or most in repose, when the hand is inclined backwards, so that the metacarpe forms a considerable angle with the forearm,* when it is also inclined inwards towards the ulnar side of the arm, and when the fingers are moderately flexed. In this case it will be perceived that the longitudinal axis of the forearm, if prolonged, would not correspond with that of the hand, but would pass through, or very near, the point, where the thumb and index finger most easily and naturally meet. Thus in the innumerable manipulations with the thumb and fingers (as with a pen, pencil, button, needle, money, &c. &c.,) their points most easily and naturally meet in this axis of the forearm. This will be found to be the position of the hand, when it hangs by the side with all the muscles relaxed.

This consideration is of little comparative importance in the case of young persons, and of those who have followed no laborious handicraft; but to persons advanced in life, and to those whose muscles and joints have become rigid by hard labor, and to whom the hand is the means of subsistence, it is a point of very material importance. A large portion of these fractures occur among such patients. When such a hand is firmly swathed by a roller upon the long straight palmar splint, it is forced into a constrained position, and some of the muscles, acting on the fragments, are put into extreme tension. This condition of the muscles must act strongly on the fragments of the radius, and must tend strongly to derange them, especially when the fracture is oblique.

*Malgaigne calls this *la flexion habituelle de la main en arrière.*

To counteract this tendency to displacement of the fragments on account of the tense condition of the muscles, the bandage with the compresses must be applied so tightly as greatly to increase the risk of that frequent ill success so well described by Dr. Barton.

When the hand is placed in the position above described, so as to take off tension from all the muscles, there will be so little tendency to displacement of the fragments, that a very gentle pressure of compresses and bandages will be adequate to maintain them in their proper relation to each other. The dressing may be removed earlier, so as to give motion to the hand and fingers, without danger of producing derangement of the fragments, and the gentle pressure of the dressing will be less likely to deprive the tendons and sheaths of their lubricity, and thus to cause permanent adhesions.

There are cases, as before observed, where such violence is done to the bones and soft parts, especially in elderly persons of a rheumatic or gouty diathesis, that it may be impossible to avoid permanent adhesions and rigidity. In such cases, if the usual authorized mode of treatment be adopted, the result will be a most awkward, unsightly, useless member.

But if the hand can be placed and retained in the unconstrained natural position above mentioned (to say nothing of the better chance of escaping permanent stiffness,) *in the first place*, the unsightly deformity will be avoided; and in the *next place*, the hand will not entirely have lost its uses. For the hand, thumb, and fingers being placed very nearly in the position of their most frequent uses, the interossei, the lumbricales, and the several short muscles of the thumb will, by causing only a very limited motion, enable the hand to perform very many of its useful functions.

I can say with confidence, not only from *à priori* reasoning, but from some experience within the last few years, that the dressing of a limb on the principles here inculcated, will very materially conduce to the comfort of a patient. I shall here make no comment upon what is said about paralyzing the muscles by tight bandages, nor upon the power of the body to accommodate itself, by a very painful discipline, to very distressing necessities.

The importance of the position of the hand in the treatment of fractures of the radius has been fully recognized for a long time by eminent surgeons. In these cases, Mr. Cline did not allow the splints or the sling to extend below the wrist. His object was to let the hand, by its own weight, and without any impediment, incline towards the ulnar edge of the forearm ; and while the ulna acted as a counter-extending force, this inclination of the hand would prevent the fragments from overriding or overlapping each other, and make it very easy to keep them in apposition. He understood well the mechanism of this accident. When the radius alone is broken, the ulna affords all requisite counter-extension ; and in proportion as the hand is inclined towards the ulna, will the lower fragments be drawn down, so that there will be hardly a chance for one fragment to overlap the other ; certainly there will be little difficulty in keeping the fragments in apposition with very gentle means. But Mr. Cline's method of dressing, in order to accomplish the indication, was too indeterminate ; he could not depend upon maintaining steadily the same degree of inclination of the hand, and one might suppose that there would be danger of producing artificial joints. Nevertheless, I am persuaded that, with Mr. Cline's method of treatment with short splints, there would be fewer cases terminating in deformity and loss of the use of the hand, than when the arm and hand are tightly swathed in long straight splints.

Sir Charles Bell long ago inculcated the importance of the inclination of the hand in the treatment of fractures of the forearm, and he has given a plate illustrating his opinion. Boyer is very explicit upon this point. He says, "the extension should be made by inclining the hand towards the ulnar edge of the forearm." Yet this obvious principle and this explicit direction are wholly disregarded in the present usual mode of dressing with long straight splints and tight bandages. Dupuytren, whose lectures on this subject should be studied by every surgeon, devised a splint—his *attelle cubitale*—with the special object of maintaining this inclination of the hand towards the ulna. Notwithstanding this great man devoted such deep attention to this subject, there were serious defects in his apparatus, which have been pointed out by subsequent French writers.

I have attempted to devise a mode of dressing these fractures, having reference to the principles advanced in this paper, and that will meet the following indications:—

1. To maintain such an inclination of the hand upon the fore-arm as shall most effectually relieve the muscles from tension, or put them in repose.

2. To maintain the hand and fingers in a position that, if rigidity should result, the member shall be as little an incumbrance, and retain as many of its uses, as possible.

3. To make it easy of application, requiring no extraordinary skill or dexterity, and little liable to be deranged or displaced.

4. To make the dressing easy and comfortable to the patient, while it does not lack efficiency.

My own experience of its use, within the last three years, convinces me that I have to some extent accomplished these indications. How far this shall be corroborated by others, can be known only when others shall have had time, opportunity, and disposition to test it.

To enable others to test the principles herein maintained, in the mode of treating these fractures, I offer the following directions for preparing the dressing, with some explanations as to its application:—

Fig. 1. A board cut to correspond with the profile of the forearm and hand.

Fig. 2. *A*. This whole space down to the *palmar block* (*B*) to be covered with pasteboard. *C*, *C*. Parts of the block, which must be more or less cut away, to suit the prominences of the hand. *D*, *D*. The projected and elevated edges of the pasteboard.

1. With a light board, of proper thickness for a splint, take a profile of the well forearm and hand of the patient, placing the hand in its habitual inclination towards the ulnar side of the arm, and extending the profile from the elbow downwards, so that it shall reach the second joint of the fingers on the inside, when these are moderately flexed—as much flexed as they are when the points of the thumb and fingers are brought into contact. The lower end of the board must be cut off obliquely (at an angle of fifteen or eighteen degrees) in a direction corresponding with that of a body grasped in the hand, when the hand is inclined to the ulna, as above indicated.

2. Cover the board thus prepared with sheeting, or other strong fabric. This may be done by winding around it, from end to end, a narrow rolling bandage, covering all of it as nearly as may be, with few or no duplications. This is the most expeditious method. A neater one is to cut a piece of sheeting, of the general form of the board, but extending beyond the board on every side, and fastening it upon the board either by a few stitches, drawing towards each other the overlapping edges, or glueing down those edges upon that side of the board which is to be towards the arm, and which edges are to be covered with the pasteboard.

3. Prepare a block of soft, light wood, from seven-eighths to eleven-eighths of an inch thick, and from two to two and a half inches wide, according to the size of the patient's hand, and of a length corresponding with the width of the board in the palm of the hand. This block is to be carved and rounded, so as to adapt it to the form of the hand, and make it easy for the thumb, and in the grasp of the hand when it is placed on the board. It is to be fastened there by screws or nails, so that the remote edge of it shall correspond exactly with the lower oblique end of the board.

4. Upon that part of the board not covered by the palm-block, fasten, by means of small carpet tacks, a piece of bookbinder's pasteboard, extending on each side beyond the edges of the board about an inch. If the pasteboard be very thick and stiff, make a slight incision in it along the edge of the board, in order to bend more easily the two projecting portions of it, thereby making a kind of box for the lodgment of the arm.

It seems to me that this splint, or one constructed on the same principles, will meet the above-mentioned indications in the following manner: *First.* The form given to the board retains the hand in its habitual inclination towards the ulnar edge of the arm, accomplishing the object aimed at by Dupuytren's *attelle cubitale*, with as much certainty, with more simplicity, and more comfort to the patient. *Second.* The palmar block retains the hand in its habitual inclination backwards, and it gives the fingers that moderate flexion which most relieves the muscles from tension, and likewise that position which, if stiffness should result, will not only save the hand from a most inconvenient, ungraceful deformity, but will reserve to it the power of performing very many of its most frequent and useful functions. In addition to these advantages, this block contributes much to the comfort of the patient. *Third.* The object in covering the board with a strong fabric, as above described, is to retain the bandage with certainty in its place, without applying it with a dangerous tightness; for, by fastening the roller to this covering with pins, the surgeon need never have his patience tried by finding his dressing deranged, at his next visit. I can speak with confidence on this point, from having used it repeatedly in cases where this quality was fully tested. *Fourth.* The pasteboard is not an essentially necessary part of the splint, but it will be found to contribute to the comfort of the patient and the convenience of the surgeon.

The requisites for dressing with this splint are flannel or other soft fabric, to cover or line the inside of the splint; two compresses; a roller; sometimes, but not always, a dorsal splint.

The flannel or other fabric with which the splint is lined should extend a little beyond the edges of the pasteboard, and the same piece may be extended over the palmar block; but it will be better to cover this block with a separate piece. For this purpose, take a piece of flannel large enough, when it is doubled, to cover the block. Through the doubled edge, with a proper needle, carry a small string (such as ligature-twine,) and tie this around the splint immediately above the block. The covering of the block thus applied may be conveniently changed, without removing the arm from its bed.

Two compresses will generally be required: the anterior or palmar, and the posterior or dorsal. The proper construction and application of the former of these are a most important point in this dressing, and certainly not less so when long, straight splints are employed; and deformity of the radius or wrist will most frequently result from negligence or want of skill in its use. If the compress be deficient in thickness, and the bandage be applied with its usual tightness, there will not fail to be either a curvature forwards, or a sigmoid flexure, which are the usual deformities. If the thickness of this compress be excessive, there may be a curvature backwards, which I think seldom occurs; but there will be such undue pressure by such a compress as to increase the danger of adhesions, and to aggravate the discomfort of the patient.

I consider this point so important that I shall run the risk of being thought tediously minute.

It has seemed to me a notable defect in Dr. Barton's mode prescribed for dressing this fracture with straight splints, that he directs the two compresses to be of equal dimensions, and of about the same dimensions in every case. He says, "Two compresses, each about two inches square, and composed of strips of bandage about one yard and a half long, evenly folded up, are to be in readiness." Dr. Barton's very eminent skill would undoubtedly vary from this direction, as each case might require. But we object to this as a rule for those who follow rules implicitly.

If we apply a long, straight splint to the palmar side of several uninjured forearms, we shall find the space between the splint and that part of the forearm, to which the compress is usually applied, to vary very much, being two or three times as great in some cases as others. This space will depend upon the form of the bones of the forearm (which are sometimes considerably curved backwards,) upon its muscularity or emaciation, and upon the greater or less prominence of the heel of the hand. This anterior compress must, therefore, vary in thickness, according to the varying form of the forearm and hand.

In order to determine with precision the requisite thickness of this compress in any case, place a long, straight splint upon the palmar side of the uninjured forearm of the patient, and make a compress of such thickness as to fill the space, so that the splint applied shall bear as firmly upon the compress as the ends of it do upon the wrist and upper part of the forearm. It is to be observed that, when the hand has its usual inclination backwards, the space between the forearm and splint will be less, and of course require a compress of less thickness, than when the hand and forearm are swathed upon a long, straight splint.

With the splint here presented to the College, it is wholly unnecessary to attach this compress to the forearm by means of a roller. And where there is not such a necessity, it is generally worse than useless to apply a roller directly to the arm before the splint is applied. Some eminent surgeon has said, that the only advantage is, that it impresses bystanders with an idea of the complexity and difficulty of the operation. If excoriation should be threatened, cover the part with soap plaster. Place this anterior compress on the splint, so that its lower edge shall approach very near to the lower end of the upper fragment of the radius, when the forearm is laid upon the compress in the splint.

These preparations being made, the fragments, if deranged, are to be reduced, and the splint applied. I have very seldom, if ever, found any difficulty in replacing the fragments. Let an assistant, grasping the hand (not the fingers), incline it towards the ulnar side of the arm, according to the direction of Boyer, making steady, but not very vigorous tension, and with his thumb and fingers the surgeon will easily press the projecting fragments into their proper relation to each other. In making this extension, Dr. Dorsay directs the assistant to "grasp the hand." Dr. Barton says an assistant "makes extension from the fingers." It seems to me that Dr. Barton errs on this point. The objections are, first, that by grasping and pulling the fingers, the flexor muscles are brought into so much more tension, that they will offer more resistance to the extending force. In the next place, by pulling at the fingers the hand will be brought more nearly into a direct line with the forearm, instead of being inclined towards the ulna, the utility of which inclination in such cases is, I think, so obvious that it would be superfluous to cite authority or to attempt a demonstration.

After the forearm is laid into the splint, apply the dorsal compress. This compress is seldom essentially necessary in these cases, but it may always be advisable to use it. Its thickness is comparatively unimportant, especially when a dorsal splint is not employed. It may be made of folds of a bandage of about the width of the wrist, and so long as to cover the lower fragment of the radius and the wrist, but not extend upon the hand. After adjusting this compress, apply a roller, beginning upon the lower fragment of the radius, carrying it down over the wrist, the metacarpus and the first joints of the fingers, leaving the thumb free; then returning with the bandage to the upper end of the splint, and attaching it in several places by pins to the woven covering of the splint. If the compresses have been properly made and adjusted, it is unnecessary, with this splint, to apply the bandage with anything like the tension ordinarily employed in dressings with the long straight splints; and those accustomed to the use of these splints will be liable to err on this point.

A dorsal splint is unnecessary, unless the fracture occur so high up that there is danger of diminishing the interosseous space between the bones of the forearm. In such a case, it is necessary. It should be so wide that the bandage will not pass upon the fragments in such a manner as to lessen the interosseous space; and it should be so long as to reach from near the elbow to the hand, but not extend upon the metacarpus.

It may sometimes be necessary to use longitudinal narrow compresses to prevent the contraction of the interosseous space. But where no roller is applied directly upon the arm, it is probable that these long narrow compresses will not commonly be found necessary.

Although the preceding observations have had an especial reference to fractures of the lower end of the radius, probably some of the principles advanced and the means proposed to carry them out, may be extended farther. How far they may be applicable to other injuries or diseases of the forearm and hand, I leave to be determined by future observation.

## Figures and Tables

**Fig. 1. f1:**
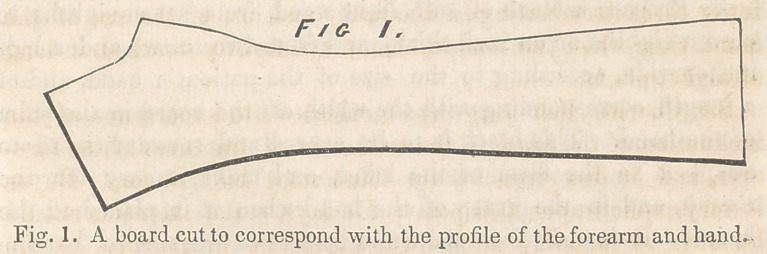


**Fig. 2. f2:**